# Seasonal Survey and Cox1-Based Molecular Characterization of Chewing Lice from Migratory Birds at the Shakpak Ornithological Station, Kazakhstan

**DOI:** 10.3390/life16091473

**Published:** 2026-09-03

**Authors:** Lazzat Ukibayeva, Andrey Gavrilov, Maratbek Suleimenov, Adnan Ayan, Laura Zhanteliyeva, Akmaral Dauletbekova, Marat Aisin, Altyn Kulpiisova, Tursungali Baitubayev, Altyn Zhubantayeva, Galiya Ibadullayeva, Leila Kassymbekova, Bazarkhan Imangaliyeva, Gulnar Altayeva, Gulzhainar Zhaksygalikyzy, Aigul Kaliyeva, Arman Issimov

**Affiliations:** 1 Department of Parasitology, Institute of Zoology, Almaty 050060, Kazakhstan; 2 Department of Zoology, Histology and Cytology, Al-Farabi Kazakh National University, Almaty 050040, Kazakhstan; 3 Department of Parasitology, Faculty of Veterinary Medicine, Aksaray University, Aksaray 68100, Türkiye; 4 Department of Parasitology, Faculty of Veterinary Medicine, Kyrgyzstan-Türkiye Manas University, Bishkek 720044, Kyrgyzstan; 5 Department of Biology and Agricultural Specialties, International Taraz University Named After Sherkhan Murtaza, Taraz 080000, Kazakhstan; 6 Department of Veterinary Medicine, A. Baitursynov Kostanay Regional University, Kostanay 110000, Kazakhstan; 7 Department of Zootechnology, Genetics and Breeding, Toraighyrov University, Pavlodar 140000, Kazakhstan; 8 Institute of Industrial Technologies, West Kazakhstan Innovation and Technology University, Oral 090000, Kazakhstan; 9 Department of Pharmacy, Kazakh National Medical University Named After S.D. Asfendiyarov, Almaty 050000, Kazakhstan; 10 Department of Chemistry and Food Technology, K. Zhubanov Aktobe Regional University, Aktobe 030000, Kazakhstan; 11 Department of Biology, K. Zhubanov Aktobe Regional University, Aktobe 030000, Kazakhstan

**Keywords:** avian ectoparasites, chewing lice, Phthiraptera, migratory birds, cox1, molecular characterization, Kazakhstan

## Abstract

Migratory birds host diverse ectoparasites, but molecular information on avian chewing lice from Central Asia remains limited. We characterized ectoparasites recovered from wild birds during spring and autumn migration at the Shakpak Ornithological Station, Kazakhstan, and molecularly characterized selected chewing lice. Field records from 20 April–10 May and 25 September–13 October 2025 were consolidated. Representative lice from four host species were photographically examined, and five selected isolates from a black kite (*Milvus migrans*), two barn swallows (*Hirundo rustica*), a European roller (*Coracias garrulus*), and a European bee-eater (*Merops apiaster*) underwent PCR amplification, bidirectional Sanger sequencing of a partial mitochondrial *cox1* fragment, and phylogenetic analysis. Among 1882 examined birds, 99 ectoparasites were recovered, comprising 88 chewing lice and 11 blood-feeding flies. Because ectoparasite counts were available only as aggregate host-taxon records and were not linked to individual birds, the field data describe specimen recovery only and cannot be used to estimate infestation prevalence, mean abundance, or mean intensity. The five sequenced isolates were tentatively assigned at genus level to *Craspedorrhynchus* sp. (BL1), *Brueelia* sp. (BL2–BL3), *Capraiella* sp. (BL4), and *Meropoecus* sp. (BL5), with GenBank accessions PZ756682–PZ756686. Photographic morphology was broadly concordant with these genus-level placements, although bootstrap support was modest (54–58%). These findings provide baseline molecular data on migratory-bird chewing lice in Kazakhstan and support expanded individual-host, slide-mounted voucher, and multilocus surveillance.

## 1. Introduction

Chewing lice are permanent, obligate ectoparasites that complete their life cycle on the host and feed primarily on feathers, skin debris or related integumentary resources. Their persistence on individual birds, frequent host specificity and differentiation among plumage microhabitats make them informative systems for studying host–parasite association, ecological specialization and coevolution [1,2]. Standardized terminology and sampling design are particularly important in avian ectoparasitology because parasite prevalence, mean abundance and mean intensity describe different biological quantities and require individual-host data [3].

The distribution of avian lice is shaped by host ecology and by opportunities for transmission. Direct contact between birds can facilitate transfer, while some feather lice disperse phoretically on blood-feeding flies. Host preening, body size, plumage structure and the louse’s microhabitat specialization can also influence detectability and persistence [4,5,6,7]. At evolutionary scales, cospeciation may contribute to host-associated diversification, but host switching and repeated ecological convergence are also widespread [8,9,10]. Migration creates an additional spatial framework: shared staging areas and flyways may promote contact among hosts, although the consequences differ among host–louse systems [11].

Reliable identification remains a major limitation in studies of chewing-louse diversity. Morphological identification requires taxonomic expertise and well-preserved voucher material, while molecular markers can assist in placing immature, damaged or morphologically ambiguous specimens. The mitochondrial cytochrome *c* oxidase subunit I gene (*cox1*) has been widely used for louse systematics and host–parasite studies [12,13,14,15,16,17]. However, mitochondrial evolution can be unusually rapid in lice, and a short single-locus fragment may not resolve closely related species or deeper relationships [15]. Molecular assignments must therefore be interpreted against reference sequence quality, phylogenetic support and morphology [18,19,20,21].

The *Brueelia* complex illustrates these challenges. Molecular evidence has demonstrated that historical generic limits conceal substantial diversity, while subsequent morphological revision established revised genera and diagnostic frameworks [12,13,22]. Regional surveys combining host records, morphology and DNA data have revealed both expected and previously unrecognized host associations [16,23]. Chewing lice of birds have been documented historically in Kazakhstan and elsewhere in Central Asia through predominantly faunistic and morphology-based studies. Groza [24] reported chewing lice from wild galliform birds in Kazakhstan, while Blagoveshchensky investigated the Mallophaga fauna of Tajikistan and Kasiev provided a comprehensive treatment of chewing lice associated with birds of Central Asia [25,26]. These studies demonstrate that chewing lice themselves are not undocumented in the region; rather, published regional information remains dominated by classical taxonomic and faunistic approaches, whereas molecular sequence-based characterization of avian chewing lice remains comparatively limited. Accordingly, the principal knowledge gap addressed by the present study concerns molecular characterization rather than the general occurrence of chewing lice in Central Asia.

The Shakpak Ornithological Station provides access to diverse birds during seasonal migration. The present study had two objectives: (i) to summarize ectoparasites recovered from birds examined during spring and autumn migration in 2025, and (ii) to identify five selected chewing-louse isolates using partial mitochondrial *cox1* sequences and phylogenetic analysis. Because the field records were aggregate rather than individual-host datasets, the study was designed as a descriptive survey and does not estimate infestation prevalence, mean intensity or mean abundance.

## 2. Materials and Methods

### 2.1. Animal Ethics

The study was approved by the Institutional Review Board and Animal Ethics Committee of the Institute of Zoology of the Science Committee of the Ministry of Higher Education and Science of the Republic of Kazakhstan (permit number: 004/025, approval date: 17 February 2025). Bird handling and ectoparasite collection were undertaken within the station’s migration-monitoring activities.

### 2.2. Study Design and Location

A descriptive field and molecular study was conducted at the Shakpak Ornithological Station, located between the Turkestan and Zhambyl regions of Kazakhstan (42°31′48′′ N, 70°36′00′′ E). Birds were examined during two migration periods: 20 April–10 May 2025 (spring) and 25 September–13 October 2025 (autumn). Field records included bird taxon, number of examined birds, and the number and broad category of recovered ectoparasites. Sex-stratified counts recorded for several taxa during spring were combined by host taxon for analysis. Duplicate or synonymous host entries were merged only when unambiguous.

Five representative chewing-louse specimens collected from the black kite (*Milvus migrans*), barn swallow *(Hirundo rustica*), European roller (*Coracias garrulus*), and European bee-eater (*Merops apiaster*) were additionally examined and photographically evaluated using an X7CAM4K16MPA 16-MP CMOS digital microscope camera (ToupTek Photonics, Hangzhou, China) mounted on a microscope imaging system at 20× magnification. The specimens were temporarily mounted for photographic examination but were not cleared or permanently slide-mounted. Images were digitally calibrated for dimensional measurement and obtained in dorsal and/or ventral orientation. Morphological assessment focused on overall body habitus, cephalic shape, body proportions, abdominal configuration, pigmentation, sclerotization, and visible setae. Because fine diagnostic characters were not uniformly resolved, photographic morphology was used as supportive phenotypic evidence rather than as an independent basis for species-level identification.

Bird and ectoparasite counts were summarized by migration period and host taxon. A descriptive specimen-recovery rate was calculated as the number of recovered ectoparasite specimens divided by the number of examined birds ×100. Because parasite counts were available only in aggregate form and were not linked to individual hosts, prevalence, mean intensity, and mean abundance were not calculated [3]. No inferential comparison between spring and autumn was performed because host composition and sampling effort differed substantially between the two migration periods.

### 2.3. Bird Examination and Ectoparasite Collection

Birds were captured as part of the routine migration-monitoring and ringing programme at the Shakpak Ornithological Station using mist nets, stationary Rybachy-type traps and, where appropriate, hand nets (Supplementary Figure S1). Mist nets were deployed mainly within shelterbelt vegetation, while the stationary Rybachy-type traps were positioned along the Shakpak Pass on the principal migration route. Captured birds were individually ringed, identified to species using standard ornithological guides, and examined before release back into the wild. Where applicable, sex, age, body mass, fat score and wing length were recorded as part of the routine ringing protocol, and capture/ringing locations were recorded using GPS.

During handling, birds were examined for external parasites. Ectoparasites were manually removed using fine forceps, recorded according to host species, and preserved in 96% ethanol at −70 °C until tested. Field records classified ectoparasites broadly as chewing lice or blood-feeding flies. Representative chewing-louse specimens were retained for photographic and molecular characterization.

### 2.4. Selection of Chewing-Louse Isolates

The molecular subset comprised one isolate from a black kite (*Milvus migrans*; BL1), two isolates from barn swallows (*Hirundo rustica*; BL2 and BL3), one isolate from a European roller (*Coracias garrulus*; BL4), and one isolate from a European bee-eater (*Merops apiaster*; BL5). These five specimens represented four avian host species. Specimens were selected purposively to maximize representation of the host taxa from which chewing lice were recovered while prioritizing well-preserved individuals with sufficient material for reliable DNA extraction and downstream PCR amplification. Two specimens were included from *H. rustica* because this host yielded the largest number of chewing lice during the survey, allowing a limited assessment of consistency among lice recovered from the same host species. The molecular subset was therefore intended to provide representative taxonomic coverage rather than a random or quantitative sample of the entire chewing-louse collection.

### 2.5. DNA Extraction and PCR Amplification

DNA was extracted from chewing-louse material using the QIAamp Fast DNA Stool Mini Kit (QIAGEN, Hilden, Germany), according to the manufacturer’s instructions. The kit was selected because its silica-membrane purification and inhibitor-removal chemistry provide purified DNA suitable for downstream PCR. For each specimen selected for molecular analysis, the entire louse was placed into the extraction tube and lysed/homogenized during DNA extraction.

A partial region of the mitochondrial *cox1* gene was amplified using primers L6625 (5′-CCGGATCCTTYTGRTTYTTYGGNCAYCC-3′) and H7005 (5′-CCGGATCCACNACRTARTANGTRTCRTG-3′), originally described by Hafner, Sudman [14]. PCR amplification was performed in a final reaction volume of 25 µL. Each reaction contained 1 µL of DNA template at approximately 100 ng/µL (approximately 100 ng DNA per reaction), 10 pmol of each primer, BioMaster HS-Taq PCR (2×) mixture, and nuclease-free water to the final volume. Amplification was performed in a SimpliAmp thermal cycler (Thermo Fisher Scientific, Waltham, MA, USA) with initial denaturation at 95 °C for 5 min; 35 cycles of 94 °C for 30 s, 55 °C for 30 s and 72 °C for 30 s; and a final extension at 72 °C for 7 min. Products were resolved in a 1% agarose gel using 1× TAE buffer. The expected amplicon was approximately 450 bp. A no-template control containing double-distilled water and a positive control containing *Brueelia domestica* DNA were included. The *B. domestica* DNA was used solely as a procedural positive control to verify successful amplification of the target cox1 region under the applied PCR conditions and was not used as a taxonomic comparator for the study specimens.

### 2.6. Sequencing

PCR products were sequenced in both directions using the amplification primers and BigDye Terminator chemistry. Each 25-µL sequencing reaction comprised 18 µL double-distilled water, 5 µL 5× buffer, 0.5 µL BigDye reagent, 0.5 µL primer and 1 µL PCR product. Sequencing was performed on an ABI 3130XL Genetic Analyzer (Applied Biosystems, Carlsbad, CA, USA). Forward and reverse reads were inspected and edited using BioEdit version 7.0 [27]. Following inspection of the forward and reverse chromatograms and removal of low-quality terminal regions, the sequences were trimmed to a common 379-bp homologous region of *cox1* for sequence comparison and phylogenetic analysis.

### 2.7. Phylogenetic Analysis

Study sequences were initially compared against the NCBI nucleotide database using BLAST (https://blast.ncbi.nlm.nih.gov/, accessed on 1 August 2026) [28]. Reference sequences were selected primarily from the closest taxonomically relevant matches that showed adequate coverage of the homologous *cox1* region, with additional representatives of the corresponding chewing-louse genera included to provide taxonomic context. Study and reference sequences were aligned using ClustalW [29] and trimmed to the common homologous region. Neighbor-Joining analysis was performed in MEGA version 11 [30] using the Kimura 2-parameter (K2P) evolutionary-distance model, with among-site rate variation modelled using a discrete Gamma distribution with five categories. Node support was evaluated using 1000 bootstrap replicates, and values ≥ 50% were displayed at the corresponding nodes [31]. *Embidopsocus* sp. OP599544 was used as the outgroup.

## 3. Results

### 3.1. Seasonal Bird Examination and Ectoparasite Recovery

In total, 1882 birds were examined across the two migration periods. The spring survey included 317 birds and yielded 65 ectoparasites, all recorded as chewing lice. The autumn survey included 1565 birds and yielded 34 ectoparasites: 23 chewing lice and 11 blood-feeding flies. Thus, the combined collection comprised 99 ectoparasites, including 88 chewing lice and 11 blood-feeding flies (Table 1). Because these counts were available only in aggregate form and were not linked to individual birds, they describe specimen recovery rather than individual-host infestation.

### 3.2. Host Taxa Associated with Recovered Ectoparasites

In spring, all recorded chewing lice were recovered from three host taxa: European roller, European bee-eater and barn swallow. Barn swallows accounted for 45 of the 65 spring chewing-louse specimens, European bee-eaters for 17 and European rollers for three. In autumn, chewing lice were recorded from black kite, barn swallow, sand martin, rook, jackdaw, carrion crow, the great/Bokhara tit entry, a lesser-whitethroat entry and European bee-eater. Blood-feeding flies were recorded from Eurasian sparrowhawk, grey wagtail, tree pipit, barn swallow and Eurasian magpie (Table 2).

### 3.3. PCR Amplification and Sequence Generation

**Figure 1:**
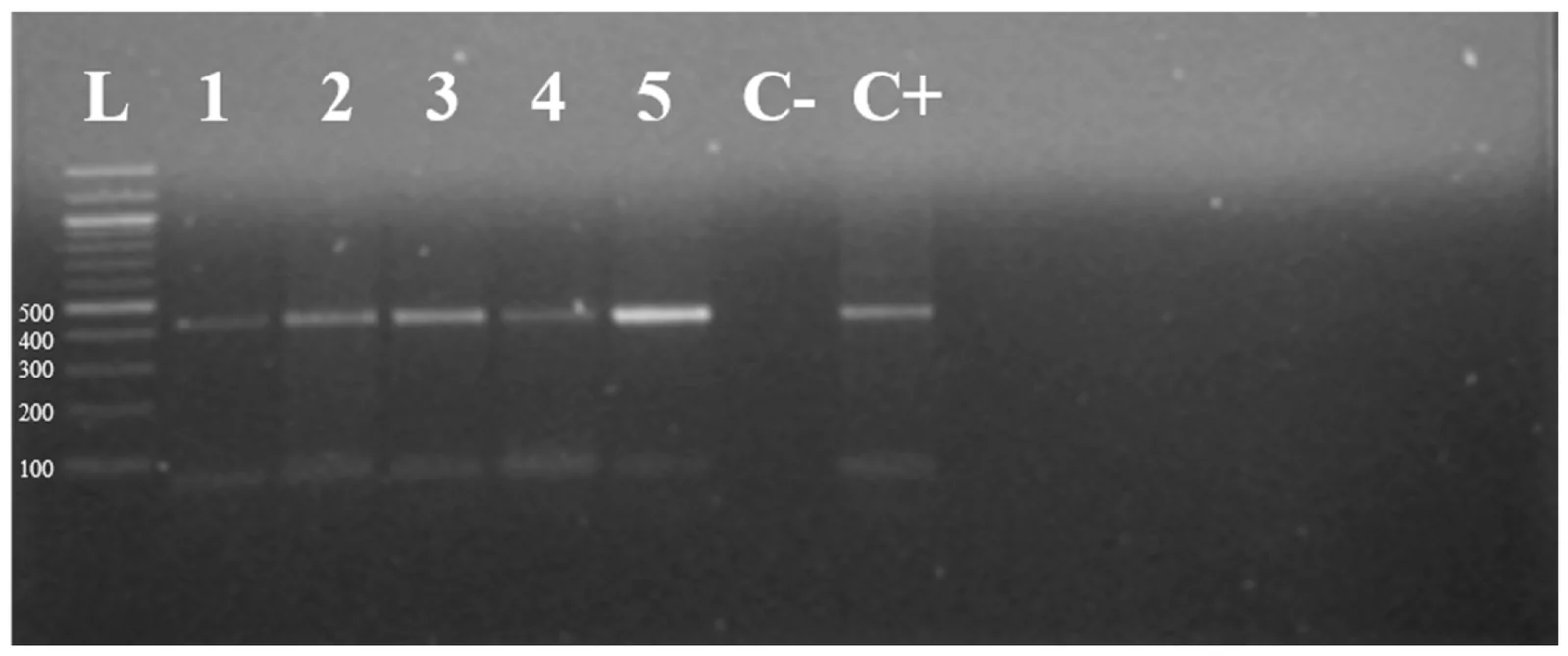
Agarose-gel electrophoresis of PCR products generated from chewing-louse DNA using *cox1* primers. L, 100–1000-bp marker; lane 1, black kite isolate; lanes 2–3, barn swallow isolates; lane 4, European roller isolate; lane 5, European bee-eater isolate; C−, no-template control (double-distilled water); C+, positive control (*Brueelia domestica*). Expected product size: approximately 450 bp.

All five selected chewing-louse samples produced visible amplicons of approximately 450 bp. The positive control produced a band of the expected size, whereas no amplification was detected in the negative control (Figure 1). The resulting products were suitable for purification and bidirectional Sanger sequencing.

### 3.4. Molecular Identification of Chewing-Louse Isolates

Sequence comparison and phylogenetic placement supported tentative genus-level placement of the five isolates within four chewing-louse genera (Table 3). BL1 from the black kite was tentatively placed within *Craspedorrhynchus* sp.; BL2 and BL3 from barn swallows within *Brueelia* sp.; BL4 from the European roller within Capraiella sp.; and BL5 from the European bee-eater within *Meropoecus* sp. The sequences were reported as deposited in GenBank under accession numbers PZ756682–PZ756686. These placements are genus-level identifications and should not be interpreted as a confirmation of named species.

### 3.5. Phylogenetic Relationships

**Figure 2:**
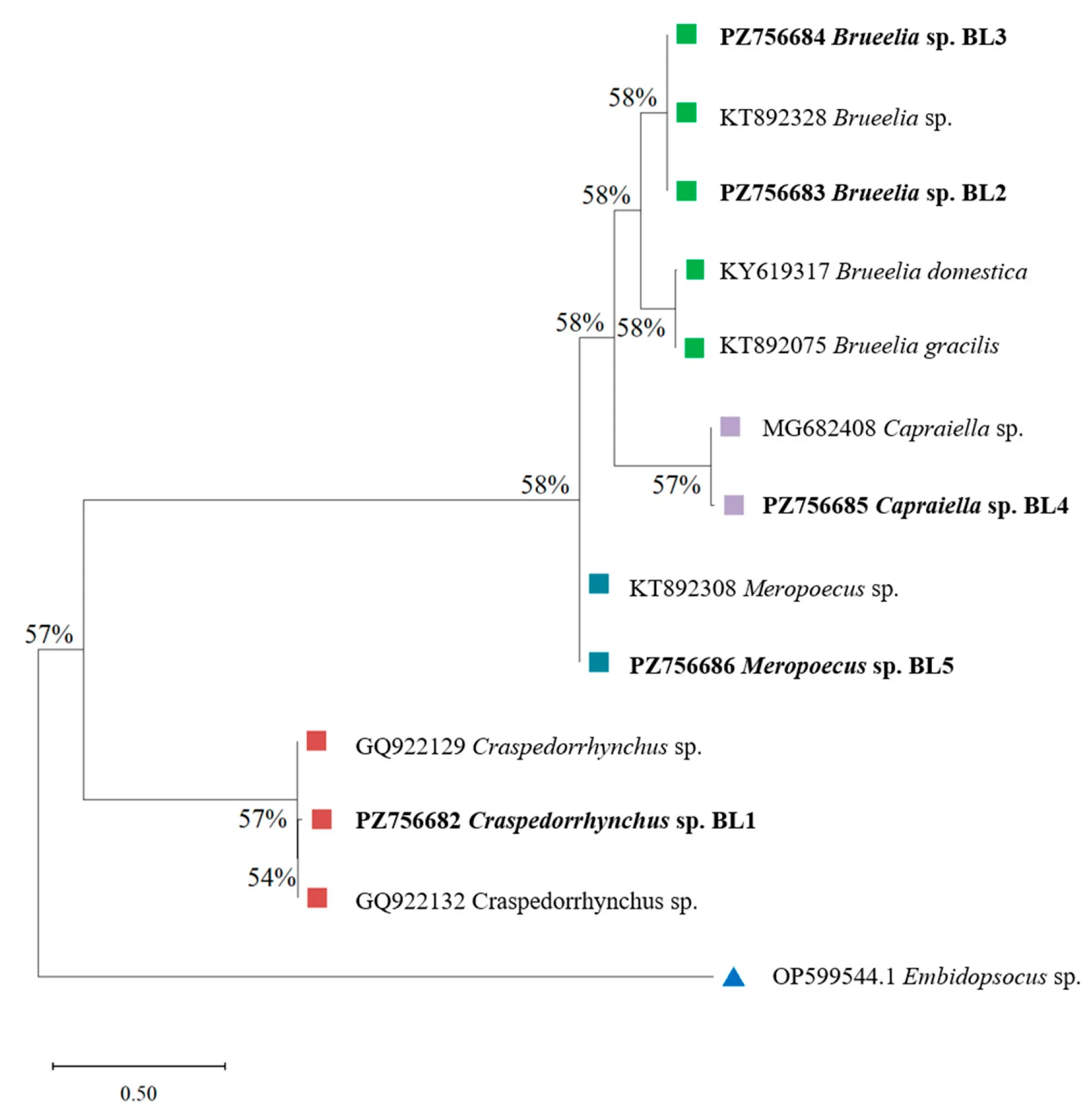
Phylogenetic tree based on partial mitochondrial *cox1* sequences of avian chewing lice. The tree was reconstructed using the Neighbor-Joining method. Node support was evaluated using 1000 bootstrap replicates, and bootstrap values ≥ 50% are shown. *Embidopsocus* sp. OP599544 was used as the outgroup. The scale bar indicates 0.50 substitutions per site.

Phylogenetic analysis separated the study specimens into four genus-associated lineages (Figure 2). Barn swallow specimens PZ756683 (BL2) and PZ756684 (BL3) clustered in the *Brueelia* lineage with *Brueelia* sp. KT892328; the broader group also included reference sequences of *B. domestica* KY619317 and *B. gracilis* KT892075. Relationships within this group received approximately 58% bootstrap support.

European roller specimen PZ756685 (BL4) clustered with *Capraiella* sp. MG682408, with 57% support. European bee-eater specimen PZ756686 (BL5) grouped with *Meropoecus* sp. KT892308 and was separated from the *Brueelia* and *Capraiella* sequences; relationships among these lineages were weakly supported, at approximately 58%. Black kite specimen PZ756682 (BL1) was placed with *Craspedorrhynchus* reference sequences GQ922129 and GQ922132. This lineage received 57% support, with internal branching supported at 54%. *Embidopsocus* sp. OP599544 was external to the chewing-louse sequences and rooted the tree. Overall, the topology was compatible with the tentative genus-level placements; however, the relatively low bootstrap support (54–58%) indicates limited resampling stability. Accordingly, these nodes were not interpreted as strong evidence for deeper relationships among the genera or for species-level identification.

### 3.6. Morphological Characterization of Recovered Chewing-Louse Specimens

**Figure 3:**
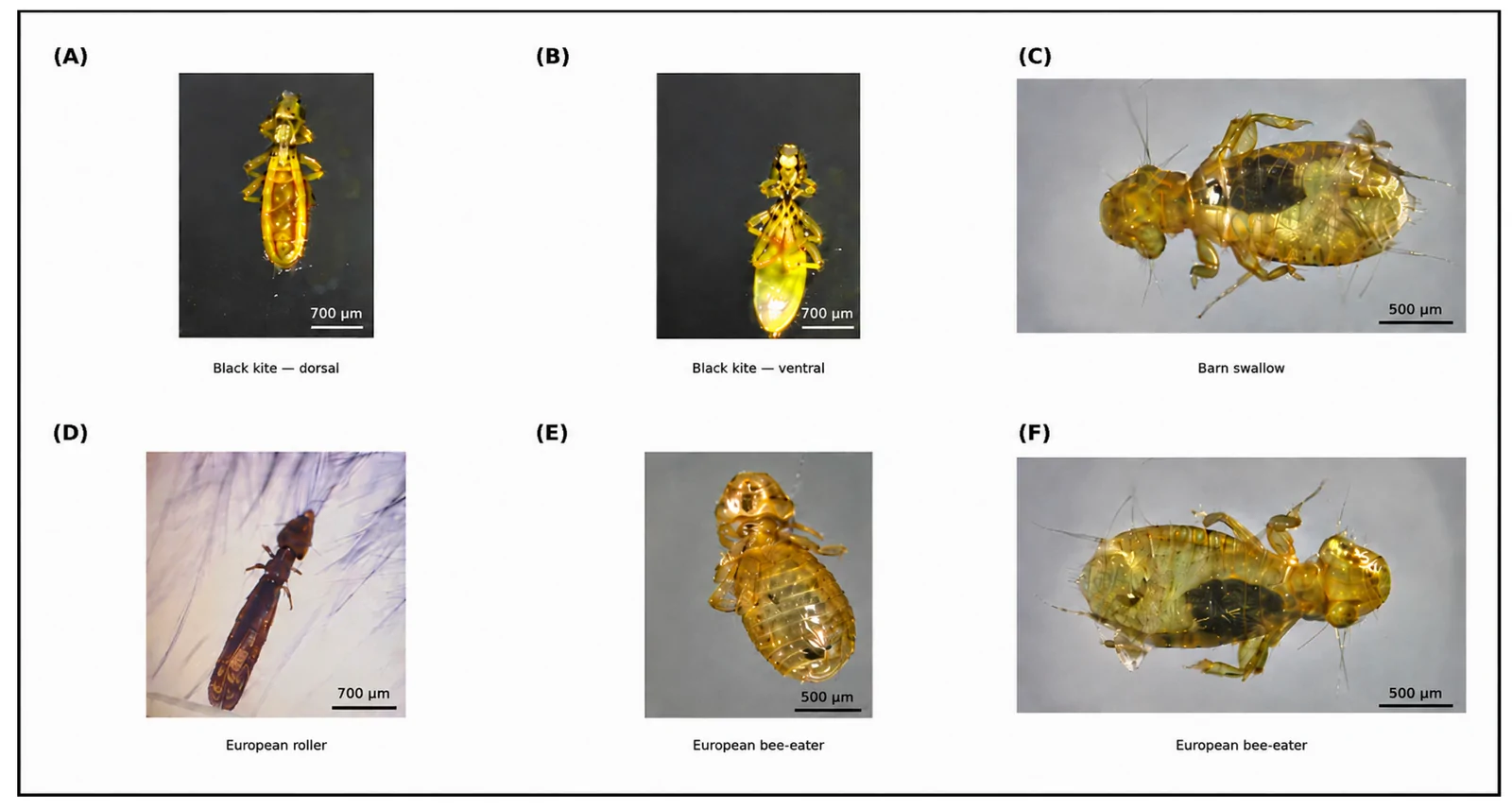
Representative morphological appearance of chewing lice collected from migratory birds at the Shakpak Ornithological Station, Kazakhstan. (**A**,**B**) Chewing louse collected from the black kite (*Milvus migrans*), shown in dorsal and ventral views, respectively; (**C**) chewing louse from the barn swallow (*Hirundo rustica*); (**D**) chewing louse from the European roller (*Coracias garrulus*); and (**E**,**F**) representative chewing lice from the European bee-eater (*Merops apiaster*). The specimens demonstrate variation in overall body habitus, cephalic configuration, body proportions, abdominal shape, pigmentation and degree of sclerotization among host-associated lice. The corresponding host groups yielded cox1-based molecular assignments to *Craspedorrhynchus* sp., *Brueelia* sp., *Capraiella* sp. and *Meropoecus* sp., respectively. Images are not shown to a common scale.

Photographic examination revealed distinct variation in body shape, cephalic configuration, pigmentation and abdominal sclerotization among chewing lice collected from the four avian host species represented in the molecular analysis (Figure 3). All representative specimens exhibited the general morphology of avian chewing lice, including a dorsoventrally flattened body, a clearly differentiated head and thorax, and a segmented abdomen.

Black-kite-associated specimens showed a relatively elongate and strongly sclerotized habitus, with a prominent cephalic region and conspicuous abdominal pigmentation and sclerotization (Figure 3A,B), broadly consistent with the molecular assignment to *Craspedorrhynchus* sp. The barn-swallow-associated specimen displayed a comparatively slender-to-oval body form with a distinct cephalic region and moderate abdominal sclerotization (Figure 3C), compatible with the molecular placement within *Brueelia* sp. The representative European roller-associated specimen exhibited an elongate body and relatively tapered anterior cephalic region (Figure 3D), supporting its placement within *Capraiella* sp. Bee-eater-associated specimens displayed a broader oval abdomen and comparatively broad cephalic region, with conspicuous abdominal segmentation and pigmentation (Figure 3E,F), consistent with the molecular assignment to *Meropoecus* sp.

Overall, the photographic morphology was broadly concordant with the genus-level assignments obtained from partial mitochondrial *cox1* sequences. However, because fine diagnostic structures were not consistently resolved, the morphological observations were considered supportive phenotypic evidence and were not used to establish species-level identification.

## 4. Discussion

The present study provides combined field, morphological, and molecular information on chewing lice associated with migratory birds examined at the Shakpak Ornithological Station in southern Kazakhstan. Across the two migration periods, 1882 birds were examined and 99 ectoparasites were recovered, comprising 88 chewing lice and 11 blood-feeding flies. Importantly, the spring and autumn chewing-louse totals (65 and 23 specimens, respectively) represent descriptive recovery counts only and should not be interpreted as evidence of a seasonal difference in infestation. Because parasite records were available as aggregate host-taxon counts rather than individual-host data, and because host composition and sampling effort differed substantially between migration periods, no epidemiological comparison of infestation between spring and autumn can be made. The field and molecular components should, however, be interpreted separately. The field survey documents the recovery of ectoparasites from a broad range of migratory birds, whereas molecular characterization was deliberately restricted to five representative chewing-louse isolates from four host species. Thus, the four genera detected by sequencing *Craspedorrhynchus*, *Brueelia*, *Capraiella*, and *Meropoecus* should not be regarded as representing the complete taxonomic diversity of the 88 chewing lice recovered during the study.

For descriptive context, chewing-louse recovery corresponded to 20.50 specimens per 100 examined birds in spring and 1.47 specimens per 100 examined birds in autumn. These values are specimen-recovery indices derived from aggregate records and do not represent prevalence, mean abundance, or mean intensity [3].

Within the spring sample, most recovered chewing lice were associated with barn swallows and European bee-eaters, which yielded 45 and 17 chewing-louse specimens, respectively. In contrast, the autumn collection was distributed across a broader range of hosts, including black kite, barn swallow, sand martin, several corvids, tits, whitethroats, and European bee-eater. Host identity, social behaviour, plumage architecture, moult and behavioural defenses such as preening can influence louse occurrence, persistence and detectability [7,32]. Differences in migration timing, age structure, moult, breeding history, and host condition may also contribute to variation in ectoparasite recovery. However, these factors were not measured specifically in the present study and should therefore be regarded as plausible explanatory mechanisms rather than demonstrated causes.

The detection of blood-feeding flies exclusively during autumn is also noteworthy. These specimens were not identified taxonomically or examined molecularly because the study design and molecular workflow were specifically focused on chewing lice; the flies were recorded only as an ancillary component of the ectoparasite collection. Blood-feeding hippoboscid flies can be ecologically relevant to feather-louse transmission because phoretic dispersal of some chewing lice on such flies has been documented [1]. Nevertheless, the present dataset does not establish any direct relationship between the flies and lice recovered from the examined birds, and the fly records should therefore be interpreted only as an additional component of the observed ectoparasite fauna.

The addition of photographic morphology provides an independent phenotypic context for interpretation of the molecular findings. Representative specimens from the four host species showed clear differences in overall body form, cephalic configuration, pigmentation, and abdominal sclerotization (Figure 3). Detailed morphology remains important because many avian ischnoceran genera are distinguished using combinations of cephalic, abdominal, chaetotaxic and genital characters, while molecular evidence can reveal convergence or cryptic diversity that is not apparent from gross morphology alone [13,20,33]. The gross morphological characteristics were broadly concordant with the genus-level assignments obtained by *cox1* sequencing. Importantly, however, the specimens were not consistently cleared and slide-mounted, and fine diagnostic characters such as detailed cephalic carinae, chaetotaxy, and genital structures could not be evaluated uniformly. Morphology was therefore used as supportive evidence rather than as a basis for independent species-level identification. This conservative approach is particularly appropriate for philopterid lice, in which closely related taxa may require detailed morphological examination and comparison with authoritative taxonomic descriptions [4,12,13].

The two barn-swallow specimens, BL2 and BL3, were both placed within *Brueelia*. This finding is biologically plausible because members of the *Brueelia* complex are widely associated with passerine hosts, and *Brueelia* spp. have previously been reported from barn swallows and other small passerines [34]. The inclusion of two specimens from *Hirundo rustica* was justified by the comparatively large number of chewing lice recovered from this host and provided a limited opportunity to assess consistency among specimens from the same host species. Both isolates were assigned to the same genus, but their placement close to an unnamed *Brueelia* reference sequence and the weak support within the corresponding clade do not justify attribution to a named species. This caution is reinforced by the substantial taxonomic complexity of the *Brueelia* complex, for which molecular analyses have revealed extensive diversity and subsequent morphological revision has redefined generic boundaries [12,13].

The European bee-eater specimen BL5 was placed within *Meropoecus* sp. and displayed a broad-bodied morphology generally compatible with this genus.

Members of *Meropoecus* are well-established ectoparasites of European bee-eaters. Previous studies have identified *Meropoecus meropis* as one of the principal chewing lice associated with *M. apiaster* and have demonstrated both horizontal transmission among adult hosts and measurable host-related effects under experimental conditions [35,36,37]. The present study, however, did not measure individual parasite burdens, body condition, reproductive performance, or other indicators of host fitness. Accordingly, no inference regarding pathological or fitness effects can be drawn from the detection of *Meropoecus* in the sampled bee-eaters. The significance of the present finding lies instead in providing molecular and photographic documentation of this host-associated lineage from Kazakhstan.

The European roller specimen BL4 was placed within *Capraiella*, while the black-kite isolate BL1 clustered within *Craspedorrhynchus*. These placements are compatible with broad host associations summarized in taxonomic references for avian chewing lice [4]. Their corresponding photographs also showed gross morphological differences consistent with the molecular separation of these specimens. Nevertheless, host identity alone cannot be used diagnostically, and neither association should be elevated to species level without examination of appropriately prepared voucher specimens and comparison with validated reference material. Integrative surveys conducted in other regions have similarly shown that combined host information, morphology, and molecular data can recover expected associations while also revealing previously unrecognized diversity [16,23].

The phylogenetic analysis placed the five study specimens within four genus-associated lineages corresponding to *Brueelia*, *Capraiella*, *Meropoecus*, and *Craspedorrhynchus*. However, these placements should be interpreted conservatively because the molecular dataset comprised only five specimens, was based on a single 379-bp cox1 region, and showed modest bootstrap support (54–58%). Accordingly, the tree is used primarily to support provisional genus-level placement rather than to infer species boundaries or deeper relationships among the genera. Broader taxon sampling, longer mitochondrial sequences, and additional independent nuclear loci would be required for more robust phylogenetic resolution.

This limitation is particularly relevant to *Brueelia*, for which broader molecular studies have shown substantial hidden diversity and complicated evolutionary relationships [12]. More generally, recent studies of feather-louse evolution increasingly employ multiple mitochondrial and nuclear loci or genome-scale datasets to obtain improved resolution of host switching, divergence, and deeper phylogenetic relationships [10,18,33]. For the Shakpak isolates, future work should combine properly slide-mounted specimens with longer mitochondrial sequences and one or more independent nuclear loci, such as EF-1α. Broader sampling of confidently identified congeners would also improve the ability to evaluate species boundaries and intraspecific diversity.

An important strength of the present study is the integration of several complementary sources of evidence. Birds were examined during both spring and autumn migration using an established capture and ringing programme, ectoparasites were linked to host taxa, representative chewing lice were photographically documented, and selected isolates were characterized using PCR, bidirectional Sanger sequencing, and phylogenetic analysis. The resulting *cox1* sequences were deposited in GenBank under accession numbers PZ756682–PZ756686. Together, these components provide a useful baseline for further investigation of avian chewing-louse diversity along a major Central Asian migration corridor.

The five-specimen molecular subset was selected purposively to maximize representation of louse-positive host taxa while retaining specimens suitable for sequencing; it was not a random or quantitatively representative sample of all 88 recovered chewing lice. Consequently, the relative frequency of the four molecularly detected genera in the complete collection cannot be estimated from these data.

Several additional limitations should be acknowledged. Most importantly, individual-host infestation data were not retained in a form permitting calculation of prevalence, mean intensity, or mean abundance. Although birds were examined during routine ringing procedures, examination time and parasite-recovery effort were not standardized specifically for quantitative ectoparasitological comparison. Seasonal differences in host composition and sample size further preclude direct statistical comparison between spring and autumn. The photographic morphology documented useful gross characters but did not consistently resolve the fine structures required for definitive species-level taxonomy. Blood-feeding flies were recorded but were not identified morphologically or molecularly. Finally, sampling was restricted to a single migration station and a single year, limiting broader geographic and temporal inference.

These limitations define clear priorities for subsequent studies. Future field surveys should assign a unique identifier to each bird and record the number and taxonomic identity of ectoparasites recovered from each individual. A standardized examination period or validated quantitative method such as dust-ruffling would permit calculation of conventional epidemiological parameters and more rigorous comparison among host species and migration periods [5,6]. Representative lice should be cleared, slide-mounted, measured, and deposited as voucher specimens in an accessible institutional collection, while high-resolution images of diagnostically important cephalic, abdominal, and genital structures should accompany molecular data. Molecular sampling should include multiple individuals from each host species and migration period and incorporate additional mitochondrial and nuclear markers. Such an integrative design would permit more reliable species delimitation, characterization of intraspecific genetic diversity, and investigation of whether migratory connectivity contributes to differentiation or homogenization of chewing-louse populations across Central Asian flyways [11,12,13].

## 5. Conclusions

This study provides baseline field, morphological, and molecular data on chewing lice associated with migratory birds at the Shakpak Ornithological Station, Kazakhstan. Among 1882 examined birds, 88 chewing lice were recovered, while five selected specimens were provisionally placed at genus level within *Craspedorrhynchus*, *Brueelia*, *Capraiella*, and *Meropoecus*. Photographic morphology was broadly consistent with these genus-level placements. However, the limited molecular subset, short single-locus sequence, and modest bootstrap support precluded confident species-level identification. Further studies using individual-host parasite records, slide-mounted voucher specimens, broader taxon sampling, and multilocus or genomic approaches are needed to clarify chewing-louse diversity and host associations along Central Asian migration routes.

## Figures and Tables

**Table 1:** Seasonal summary of examined birds and recovered ectoparasites at the Shakpak Ornithological Station, 2025.

Migration Period	Birds Examined	Chewing Lice	Blood-Feeding Flies	All Ectoparasites	Chewing Lice Per 100 Birds
Spring	317	65	0	65	20.50
Autumn	1565	23	11	34	1.47
Total	1882	88	11	99	4.68

Note: Values per 100 birds are specimen-recovery rates calculated from aggregate counts, not prevalence, mean abundance or mean intensity.

**Table 2:** Host taxa from which ectoparasites were recorded during spring and autumn migration.

Season	Host Taxon	Birds Examined	Chewing Lice	Blood-Feeding Flies
Spring	European roller, *Coracias garrulus*	7	3	0
Spring	European bee-eater, *Merops apiaster*	34	17	0
Spring	Barn swallow, *Hirundo rustica*	241	45	0
Autumn	Eurasian sparrowhawk, *Accipiter nisus*	16	0	3
Autumn	Black kite, *Milvus migrans*	3	3	0
Autumn	Grey wagtail, *Motacilla cinerea*	1	0	1
Autumn	Tree pipit, *Anthus trivialis*	1	0	1
Autumn	Barn swallow, *Hirundo rustica*	982	8	5
Autumn	Sand martin, *Riparia riparia*	38	1	0
Autumn	Rook, *Corvus frugilegus*	183	5	0
Autumn	Jackdaw, *Coloeus monedula*	41	1	0
Autumn	Eurasian magpie, *Pica pica*	1	0	1
Autumn	Carrion crow, *Corvus corone*	2	1	0
Autumn	Great/Bokhara tit (unresolved), *Parus major*/*P. bokharensis*	25	1	0
Autumn	Lesser whitethroat, *Curruca curruca*	3	2	0
Autumn	European bee-eater, *Merops apiaster*	21	1	0

Note: The table reports host groups with at least one ectoparasite. Complete host lists are provided in Supplementary Tables S1 and S2. Taxonomic wording follows or cautiously standardizes the field records. Taxonomic wording follows or cautiously standardizes the original field records. Ambiguous identifications were retained without retrospective reassignment; in particular, the Great/Bokhara tit record could not be resolved confidently between *Parus major* and *P. bokharensis* and is therefore presented as an unresolved field identification.

**Table 3:** Tentative molecular placement of selected chewing-louse specimens based on partial mitochondrial *cox1* sequences.

Specimens	Genus	Closest BLASTn Match	Reference Accession	Query Coverage (%)	Identity (%)
BL1	*Craspedorrhynchus* sp.	*Craspedorrhynchus* sp.	GQ922129	99	96.6
BL2	*Brueelia* sp.	*Brueelia* sp.	KT892328	100	98.2
BL3	*Brueelia* sp.	*Brueelia* sp.	KT892328	100	97.9
BL4	*Capraiella* sp.	*Capraiella* sp.	MG682408	99	96.8
BL5	*Meropoecus* sp.	*Meropoecus* sp.	KT892308	100	98.4

## Data Availability

The field data supporting the findings of this study are included in the article and Supplementary Tables S1 and S2. The partial mitochondrial cox1 sequences generated in this study are available in GenBank under accession numbers PZ756682–PZ756686. Additional information is available from the corresponding author upon reasonable request.

## Supplementary Materials

The following supporting information can be downloaded at: https://www.mdpi.com/article/10.3390/life16091473/s1, Table S1: Spring 2025 bird examination and ectoparasite records, standardized from the supplied field table; Table S2: Autumn 2025 bird examination and ectoparasite records, standardized from the supplied field table; Figure S1: Bird-capture equipment used during migration monitoring at the Shakpak Ornithological Station, Kazakhstan.
