# Supplementary material for: Seasonal Survey and Cox1-Based Molecular Characterization of Chewing Lice from Migratory Birds at the Shakpak Ornithological Station, Kazakhstan
Source: Life (Basel). 2026 Sep 3;16(9):1473. doi: 10.3390/life16091473 (PMC13608492; doi:10.3390/life16091473)
Supplement: Supplementary file 1 [file life-16-01473-s001.zip › life-4518704-supplementary.pdf]

Supplementary material

Supplementary Table S1. Spring 2025 bird examination and ectoparasite records, standardized from the supplied field table.

Order	Family	Host taxon	Birds examined	Chewing lice	Blood-feeding flies
Accipitriformes	Accipitridae	Eurasian sparrowhawk, *Accipiter nisus*	3	0	0
Accipitriformes	Accipitridae	Western marsh harrier, *Circus aeruginosus*	1	0	0
Strigiformes	Strigidae	Eurasian scops owl, *Otus scops*	9	0	0
Strigiformes	Strigidae	Long-eared owl, *Asio otus*	1	0	0
Coraciiformes	Coraciidae	European roller, *Coracias garrulus*	7	3	0
Coraciiformes	Meropidae	European bee-eater, *Merops apiaster*	34	17	0
Columbiformes	Columbidae	Oriental turtle dove, *Streptopelia orientalis*	2	0	0
Galliformes	Phasianidae	Grey partridge, *Perdix perdix*	1	0	0
Passeriformes	Turdidae	Song thrush, *Turdus philomelos*	1	0	0
Passeriformes	Turdidae	Black-throated thrush, *Turdus atrogularis*	1	0	0
Passeriformes	Sturnidae	Common myna, *Acridotheres tristis*	1	0	0
Passeriformes	Emberizidae	Corn bunting, *Emberiza calandra*	5	0	0
Passeriformes	Hirundinidae	Barn swallow, *Hirundo rustica*	241	45	0
Passeriformes	Hirundinidae	Pale sand martin, *Riparia diluta*	7	0	0
Passeriformes	Hirundinidae	Red-rumped swallow, *Cecropis daurica*	1	0	0
Passeriformes	Hirundinidae	Sand martin, *Riparia riparia*	2	0	0
Total			317	65	0

Reconciliation note: Sex-specific counts were combined, and duplicate entries were merged only where taxonomic identity was unambiguous. The European roller record was retained as n = 7, consistent with the consolidated spring dataset and reported seasonal total of 317 birds.

Supplementary Table S2. Autumn 2025 bird examination and ectoparasite records, standardized from the supplied field table.

**Order**	**Family**	**Host taxon**	**Birds examined**	**Chewing lice**	**Blood-feeding flies**
Accipitriformes	Accipitridae	Eurasian sparrowhawk, *Accipiter nisus*	16	0	3
Accipitriformes	Accipitridae	Black kite, *Milvus migrans*	3	3	0
Accipitriformes	Accipitridae	Hen harrier, *Circus cyaneus*	2	0	0
Accipitriformes	Accipitridae	Long-legged buzzard, *Buteo rufinus*	1	0	0
Falconiformes	Falconidae	Eurasian hobby, *Falco subbuteo*	1	0	0
Falconiformes	Falconidae	Common kestrel, *Falco tinnunculus*	1	0	0
Falconiformes	Falconidae	Lesser kestrel, *Falco naumanni*	1	0	0
Passeriformes	Motacillidae	Grey wagtail, *Motacilla cinerea*	1	0	1
Passeriformes	Motacillidae	Tree pipit, *Anthus trivialis*	1	0	1
Passeriformes	Hirundinidae	Barn swallow, *Hirundo rustica*	982	8	5
Passeriformes	Hirundinidae	Sand martin, *Riparia riparia*	38	1	0
Passeriformes	Hirundinidae	Pale sand martin, *Riparia diluta*	22	0	0
Passeriformes	Corvidae	Rook, *Corvus frugilegus*	183	5	0
Passeriformes	Corvidae	Jackdaw, *Corvus/Coloeus monedula*	41	1	0
Passeriformes	Corvidae	Eurasian magpie, *Pica pica*	1	0	1
Passeriformes	Corvidae	Carrion crow, *Corvus corone*	2	1	0
Passeriformes	Paridae	Great/Bokhara tit entry, *Parus major/P. bokharensis*	25	1	0
Passeriformes	Sylviidae	Lesser whitethroat entry, *Curruca curruca*	3	2	0
Passeriformes	Sylviidae	Second whitethroat entry as recorded, *Sylvia curruca*	34	0	0
Passeriformes	Phylloscopidae	Common chiffchaff, *Phylloscopus collybita*	28	0	0
Passeriformes	Phylloscopidae	Hume’s leaf warbler, *Phylloscopus humei*	6	0	0

Passeriformes	Fringillidae	Common chaffinch, *Fringilla coelebs*	78	0	0
Passeriformes	Fringillidae	Brambling, *Fringilla montifringilla*	30	0	0
Passeriformes	Acrocephalidae	Blyth's reed warbler, *Acrocephalus dumetorum*	20	0	0
Passeriformes	Emberizidae	Pine bunting, *Emberiza leucocephalos*	17	0	0
Galliformes	Phasianidae	Common pheasant, *Phasianus colchicus*	1	0	0
Galliformes	Phasianidae	Daurian partridge, *Perdix dauurica*	2	0	0
Columbiformes	Columbidae	Rock pigeon, *Columba livia*	1	0	0
Columbiformes	Columbidae	Common wood pigeon, *Columba palumbus*	1	0	0
Columbiformes	Columbidae	Oriental turtle dove, *Streptopelia orientalis*	1	0	0
Caprimulgiformes	Caprimulgidae	European nightjar, *Caprimulgus europaeus*	1	0	0
Coraciiformes	Meropidae	European bee-eater, *Merops apiaster*	21	1	0
Total			1,565	23	11

Note: The two whitethroat entries are reproduced separately because the source table assigns different Russian common names but overlapping scientific terminology. Their identities should be confirmed against the primary bird log. The standardized rows sum to the reported autumn total of 1,565 birds, 23 chewing lice and 11 blood-feeding flies.

Supplementary Figure S1. Bird-capture equipment used during migration monitoring at the Shakpak Ornithological Station, Kazakhstan.

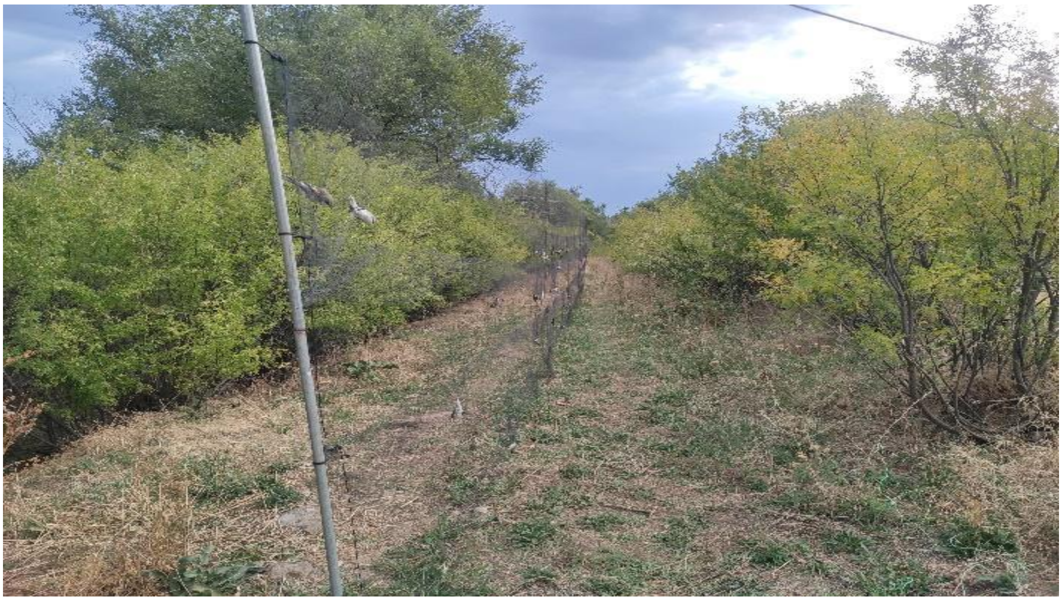

(A) Mist net deployed in shelterbelt vegetation for the capture of migratory birds;

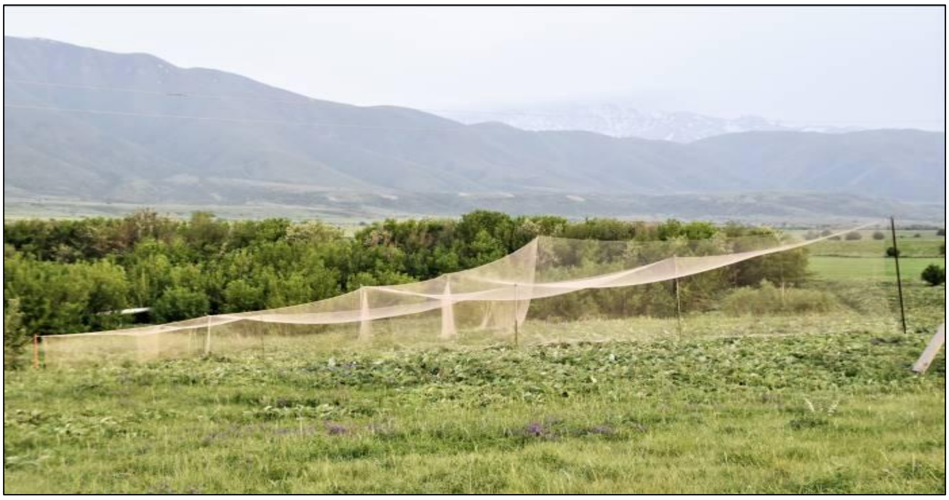

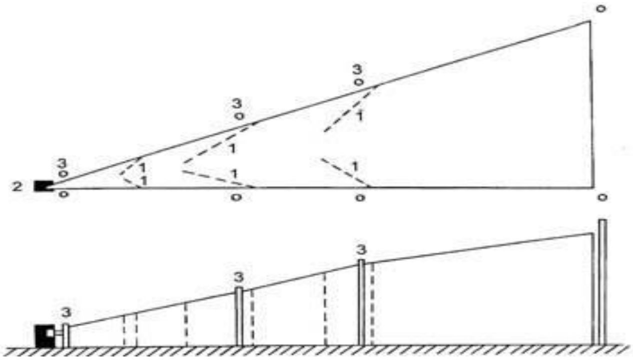

(B) schematic representation of the large stationary Rybachy-type trap used at the Shakpak Pass. The trap consists of a wide entrance leading into a progressively narrowing corridor and receiving chamber, allowing captured birds to be safely removed for ringing, morphometric examination, ectoparasite inspection and subsequent release.
